# Glucotoxicity Activation of IL6 and IL11 and Subsequent Induction of Fibrosis May Be Involved in the Pathogenesis of Islet Dysfunction

**DOI:** 10.3389/fmolb.2021.708127

**Published:** 2021-08-23

**Authors:** Liqin Lu, Lili Zhuang, Ximei Shen, Liyong Yang

**Affiliations:** ^1^ Endocrinology Department, The First Affifiliated Hospital of Fujian Medical University, Fuzhou, China; ^2^ Diabetes Research Institute of Fujian Province, Fuzhou, China

**Keywords:** T2DM, islet dysfunction, WGCNA, IL6, IL11, glycotoxicity, fibrosis

## Abstract

**Background:** Islet dysfunction is the main pathological process of type 2 diabetes mellitus (T2DM). Fibrosis causes islet dysfunction, but the current mechanism is still unclear. Here, bioinformatics analysis identified gene clusters closely related to T2DM and differentially expressed genes related to fibrosis, and animal models verified the roles of these genes.

**Methods:** Human islet transcriptomic datasets were obtained from the Gene Expression Omnibus (GEO), and weighted gene coexpression network analysis (WGCNA) was applied to screen the key gene modules related to T2DM and analyze the correlations between the modules and clinical characteristics. Enrichment analysis was performed to identify the functions and pathways of the key module genes. WGCNA, protein-protein interaction (PPI) analysis and receiver operating characteristic (ROC) curve analysis were used to screen the hub genes. The hub genes were verified in another GEO dataset, the islets of high-fat diet (HFD)-fed Sprague-Dawley rats were observed by H&E and Masson’s trichrome staining, the fibrotic proteins were verified by immunofluorescence, and the hub genes were tested by immunohistochemistry.

**Results:** The top 5,000 genes were selected according to the median absolute deviation, and 18 modules were analyzed. The yellow module was highly associated with T2DM, and its positive correlation with glycated hemoglobin (HbA1c) was significantly stronger than that with body mass index (BMI). Enrichment analysis revealed that extracellular matrix organization, the collagen-containing extracellular matrix and cytokine−cytokine receptor interaction might influence T2DM progression. The top three hub genes, interleukin 6 (IL6), IL11 and prostaglandin-endoperoxide synthase 2 (PTGS2), showed upregulated expression in T2DM. In the validation dataset, IL6, IL11, and PTGS2 levels were upregulated in T2DM, and IL6 and PTGS2 expression was positively correlated with HbA1c and BMI; however, IL11 was positively correlated only with HbA1c. In HFD-fed Sprague-Dawley rats, the positive of IL6 and IL11 in islets was stronger, but PTGS2 expression was not significantly altered. The extent of fibrosis, irregular cellular arrangement and positive actin alpha 2 (ACTA2) staining in islets was significantly greater in HFD-fed rats than in normal diet-fed rats.

**Conclusion:** Glucotoxicity is a major factor leading to increased IL6 and IL11 expression, and IL6-and IL11-induced fibrosis might be involved in islet dysfunction.

## Introduction

The main feature of diabetes is an abnormal increase in blood glucose. At present, more than 415 million people live with diabetes worldwide (Chatterjee et al., 2017). Type 2 diabetes mellitus (T2DM) accounts for more than 90% of diabetes cases and leads to microvascular and macrovascular complications that cause profound psychological and physical distress to both patients and caregivers (Chatterjee et al., 2017; Zheng et al., 2018). Although knowledge about diabetes is expanding and preventive measures are gradually improving, the incidence of diabetes continues to increase. The pathological changes of T2DM manifest mainly with insulin resistance in peripheral tissues and islet dysfunction (Kahn et al., 2014; Javeed and Matveyenko, 2018). Glucotoxicity, lipotoxicity and glucolipotoxicity are key factors leading to islet dysfunction (Lytrivi et al., 2020; Weir, 2020), and related studies have found that fibrosis is another important pathogenic factor of islet dysfunction (Homo-Delarche et al., 2006; Yang et al., 2020); however, the specific pathogenesis needs to be further clarified.

The main manifestations of glucotoxicity and lipotoxicity are abnormally elevated blood glucose and blood lipid levels, which are important causes of T2DM (Javeed and Matveyenko, 2018; Weir, 2020). Obesity (BMI ≥ 28 kg/m^2^) is positively correlated with the levels of blood lipids (Cong et al., 2014; Zheng et al., 2018). Additionally, HbA1c was positively correlated with average blood glucose, fasting blood glucose (FBG) and postprandial blood glucose levels (Monnier et al., 2003; Giugliano et al., 2008). Thus, to a certain extent, BMI and HbA1c can indirectly reflect the levels of blood glucose and blood lipids. However, in a previous study, some analyses demonstrated that the differentially expressed genes in the islets of T2DM patients were correlated with HbA1c (Mahdi et al., 2012; Taneera et al., 2012). Nevertheless, most studies have focused only on the impact of a single gene on diseases and have failed to pay attention to the influences of gene clusters. In addition, because there are not enough human islet specimens, most research results have been derived from animal models, so the mechanism of islet dysfunction has not been able to be fully clarified. Therefore, exploring the underlying pathogenesis with human specimens and gene clusters will be meaningful and beneficial for the diagnosis, treatment and prevention of diabetes.

With the application of next-generation sequencing technology, a large number of high-quality sequences of human specimens have been submitted to and stored in public databases, including the Gene Expression Omnibus (GEO), and shared with researchers around the world for free (Barrett et al., 2013). This sequencing information is convenient, useful and informative for integration and analysis of the pathological mechanism of T2DM. In addition, weighted gene coexpression network analysis (WGCNA) is a new systems biology method that converts genes into coexpression modules and network signals and has been increasingly used to discover the relationships among networks, genes and phenotypes (Wang et al., 2020). In this study, we identified the gene module with the greatest correlation with T2DM and investigated the relationship between this gene module and clinical characteristics of T2DM using WGCNA. Gene Ontology (GO) enrichment and Kyoto Encyclopedia of Genes and Genomes (KEGG) pathway analyses were further utilized to identify possible functions of key modules. Finally, WGCNA, protein–protein interaction (PPI) analysis and receiver operating characteristic (ROC) curve analysis were combined to screen hub genes, and the hub genes were verified using another database and T2DM animal models. Fibrotic proteins were detected in T2DM animal models. This study lays a foundation for exploring the potential pathogenesis and treatment of T2DM.

## Materials and Methods

### Data Collection and Preprocessing

Islet microarray data of T2DM patients were obtained from the GEO. The R/Bioconductor package Geoquery (Davis and Meltzer, 2007) was used to extract GEO objects, each of which consisted of a gene expression matrix, clinical characteristics (including age, BMI, and HbA1c) and a probe set; the expression values from the datasets were log2-transformed if necessary. The subsequent analyses were conducted on the GEO objects. The GSE41762 dataset (Mahdi et al., 2012) contained 77 islet samples from individuals (including 57 nondiabetic [ND] and 20 diabetic individuals), we eliminated 14 HbA1c deficient ND samples, therefore there were 43 ND samples left for subsequent WGCNA. (Supplementary Table S1).

### Construction of the WGCNA Network

The data were processed using the WGCNA package in R Studio 4.0.1 software. The network construction procedure included several main steps. To ensure the reliability of the results, abnormal samples were removed. The top 5,000 genes were selected on the basis of the median absolute deviation. The soft threshold for network construction was selected, and the similarity matrix was defined. The scale-free network was constructed using the blockwise module function, after which module partition analysis was performed to identify the gene coexpression modules. The adjacency matrix was transformed into a topological overlap matrix (TOM), and the corresponding dissimilarity TOM (dissTOM) was calculated. Dynamic tree cutting was performed to identify the modules from the hierarchical clusters and to calculate the module eigengenes (MEs) of each module. The MEs represented their respective modules and were used to calculate the correlations of the modules with clinical traits. The gene significance (GS) was defined as the log10-converted *p* value (GS = logP) in the linear regression between gene expression and clinical characteristics, and we defined the correlation of the gene expression profile with MEs as a module membership (MM).

### Functional Enrichment Analysis

GO enrichment and KEGG pathway analyses for the above module genes were conducted utilizing the R package clusterProfiler (Yu et al., 2012). The GO terms and KEGG pathways with *p* values < 0.05 were considered to be significant, and the top 10 were plotted using the R package GOplot.

### Identification of Hub Genes

Hub genes were screened according to the threshold values of IMMI≥0.8 and IGSI≥0.2 in the module. To construct a PPI network for the hub genes in the above modules, we further screened the hub genes using the STRING database (https://www.string-db.org/). A confidence score >0.4 was set as the threshold for selection. Cytoscape (version 3.8.2) was used to create a PPI network diagram. In this study, those with high degrees were considered to potentially represent essential genes that might contribute to T2DM. In addition, the expression levels of hub genes were compared between the T2DM group and the ND group.

### ROC Analysis

The hub genes were further screened by ROC curve analysis in OmicShare (www.omicshare.com/tools). The area under the curve (AUC) values of the hub genes were calculated and used to distinguish T2DM samples from ND samples. Generally, an AUC value ≥0.7 was regarded as a distinguishing value.

### Validation of Hub Genes in a Dataset

Dataset GSE50397 contained 77 islet samples (Fadista et al., 2014) was obtained as described in *Data Collection and Preprocessing*. Because this dataset does not include information on disease status, patients with HbA1c values ≥6.5% were considered to have diabetes according to the criteria of the International Expert Committee of the American Diabetes Association (American Diabetes Association., 2019). Finally, the expression levels of the hub genes were compared between the two groups. The R packages ggplot2, ggpubr and ggExtra were used to draw scatter plots of the correlations among the hub genes, BMI and HbA1c. Correlation analyses were performed, and the correlation coefficients were calculated. A correlation coefficient greater than 0 was considered to indicate a positive correlation; otherwise, the correlation was considered negative. *p* values < 0.05 were considered significant. ROC curve analysis of the hub genes was performed as described in *ROC Analysis*.

### Animals

#### Rats

Thirty specific pathogen-free (SPF) male Sprague-Dawley (SD) rats aged 5–6 weeks with a body weight of 220 ± 10 g were weighed and measured after 1 week of adaptive feeding (Animal Experimental Center of Fujian Medical University, China). Afterwards, the SD rats were randomly divided into two groups: 1) the normal control diet (NC) group (n = 6) and 2) the high-fat diet (HFD) group (n = 6). Body weight and body length were measured at 16, 24, 27, and 32 weeks. The model was considered successfully established when the average body weight of the HFD group was 15% higher than that of the NC group and the Lee's index value of the HFD group was 1.5% higher than that of the NC group. The Lee's index formula was $3\sqrt{(\text{weight}\times 1000)}$ /length.

#### Detection of Blood Biochemical Parameters

Rat blood samples were collected, and the supernatants were collected after centrifugation at 3,000 r/min for 10 min. FBG, triglyceride (TG), total cholesterol (TCH) and low-density lipoprotein (LDL) levels were detected by using an automated biochemical analyzer (Siemens ADVIA 2400, Germany) in the Department of Clinical Laboratory at the First Affiliated Hospital of Fujian Medical University.

#### Hematoxylin and Eosin and Masson Staining

Pancreatic tissue was soaked in 4% paraformaldehyde for 4 h and then embedded in paraffin. Then, H&E staining and Masson staining were performed, and the tissues were scanned with a pathological biopsy scanner (Pannoramic DESK/P-MIDI/P250, Hungary). Images were analysed with ImageJ software (http://imagej.nih. gov/ij/).

#### Immunohistochemistry

After dehydration, paraffin-embedded tissues were treated with citric acid antigen retrieval solution for antigen repair, 3% H_2_O_2_ was used to block endogenous peroxidase, and further serum blocking was performed. Next, the tissues were incubated using anti-IL6 antibodies (1:500 dilution, Servicebio), anti-IL11 antibodies (1:200 dilution, Thermo Fisher) and anti-PTGS2 antibodies (1:200 dilution, Immunoway). Subsequently, the tissues were incubated with secondary antibodies (HRP-labeled, Servicebio) and developed with DAB (Servicebio). The nuclei were counterstained with hematoxylin, and the samples were dehydrated, mounted and observed under a microscope. The positive staining was brown. The pancreatic islets in each slice were observed in high-power fields. All images were analysed with ImageJ software.

#### Immunofluorescence Staining

After dehydration of rat pancreatic paraffin sections, antigen repair was performed with EDTA antigenic repair solution (pH 8.0). The sections were then washed with PBS (pH 7.4) and blocked in serum. After the blocking solution was removed, the sections were incubated with ACTA2 antibodies (1:200 dilution, Abways Technology) overnight in a wet box at 4°C. After washing, the sections were incubated with secondary antibodies at room temperature in the dark for 50 min. Then, the nuclei were restained with DAPI. The sections were washed, incubated with autofluorescence quenching agent for 5 min and washed with water for 10 min. Finally, the slides were sealed with anti-fluorescence quenching agent and then observed and imaged under a fluorescence microscope. ImageJ software were used here to analyze the red fluorescence in the islet.

### Statistical Analysis

The data were processed with GraphPad Prism eight and are expressed as the mean ± standard deviation. Independent sample T-tests were used for comparisons between the two groups. When the data conformed to a normal distribution and the variance was heterogeneous, the Welch correction in the *t*-test was used. When the data did not fit a normal distribution, the Kruskal-Wallis test was used. *p* < 0.05 was considered to indicate statistical significance.

## Results

### WGCNA Network Construction

**FIGURE 1:**
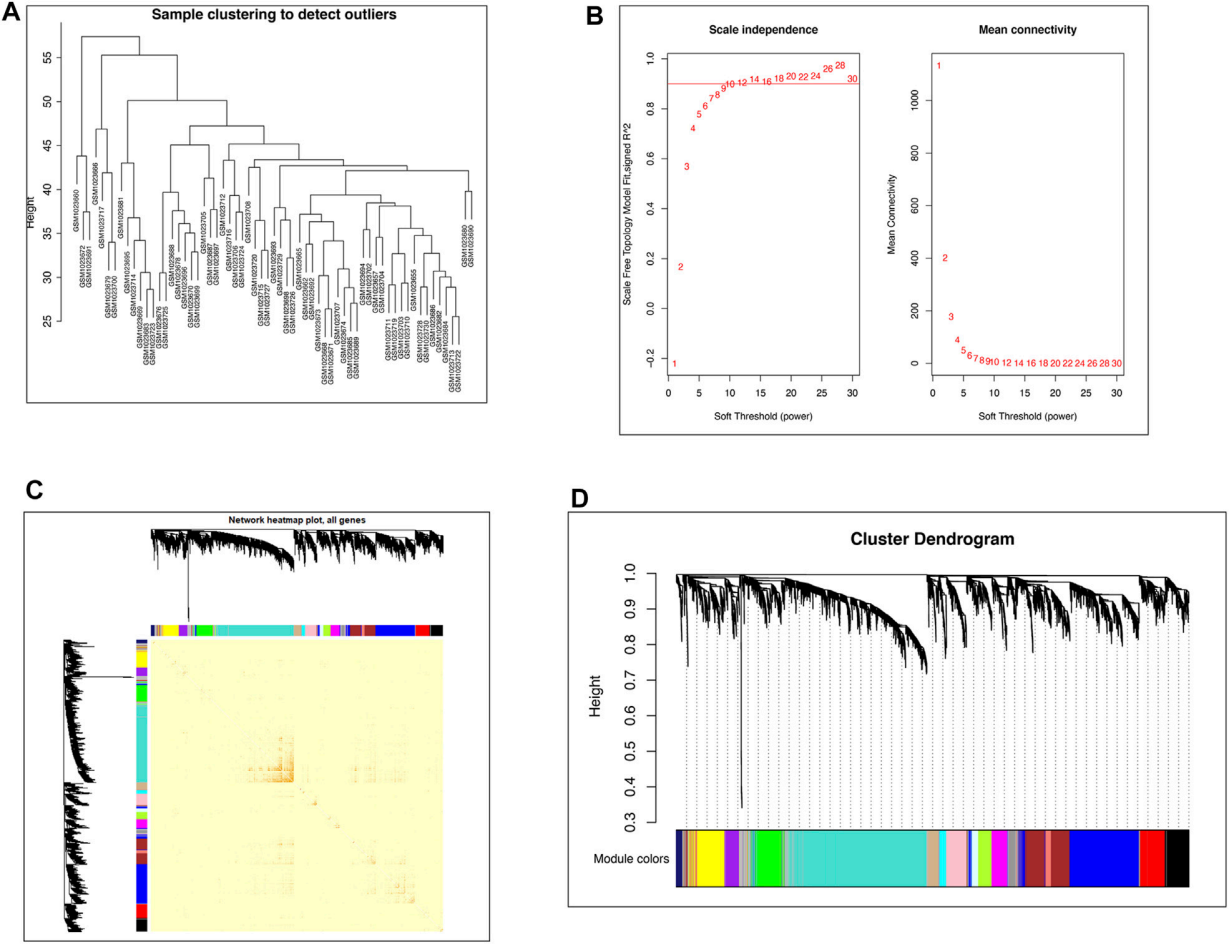
WGCNA network construction. **(A)** Clustering dendrogram of 63 samples to detect outliers. **(B)** Network topology analysis under different soft thresholds. Left: Impact of the soft-threshold power on the scale-free topology fit index. Right: Analysis of the mean connectivity for various soft-threshold powers. The power selected was 12. **(C)** Visualized gene network of the heatmap plot. A darker red color indicates greater overlap, and a light color indicates low overlap. The blocks of darker color along the diagonal are the gene modules. The module assignment gene and dendrograms are on the top and left, respectively. **(D)** Gene clustering dendrogram obtained by hierarchical clustering on the basis of adjacency-based dissimilarity.

The top 5,000 genes according to the median absolute deviation in GSE41762 were used to construct the network for WGCNA. All 63 samples were retained after the outlier samples were eliminated (Figure 1A). When the soft threshold was 12, the scale-free topology index was 0.9, and the resulting network was closer to the real biological network (Wang et al., 2020) than when other parameters were used (Figure 1B). Then, the dissTOM was obtained and subjected to hierarchical clustering, resulting in a hierarchical clustering tree (Figure 1C). According to the dynamic tree cutting method, the minimum number of genes in each module was defined as 50, a mid-level classification (deepSplit = 2) was selected to identify the key clusters, and the mergeCutHeight value 0.3. Then, a total of 18 modules were ultimately obtained (Figure 1D).

### Identification of Key Clinically Significant Modules

**FIGURE 2:**
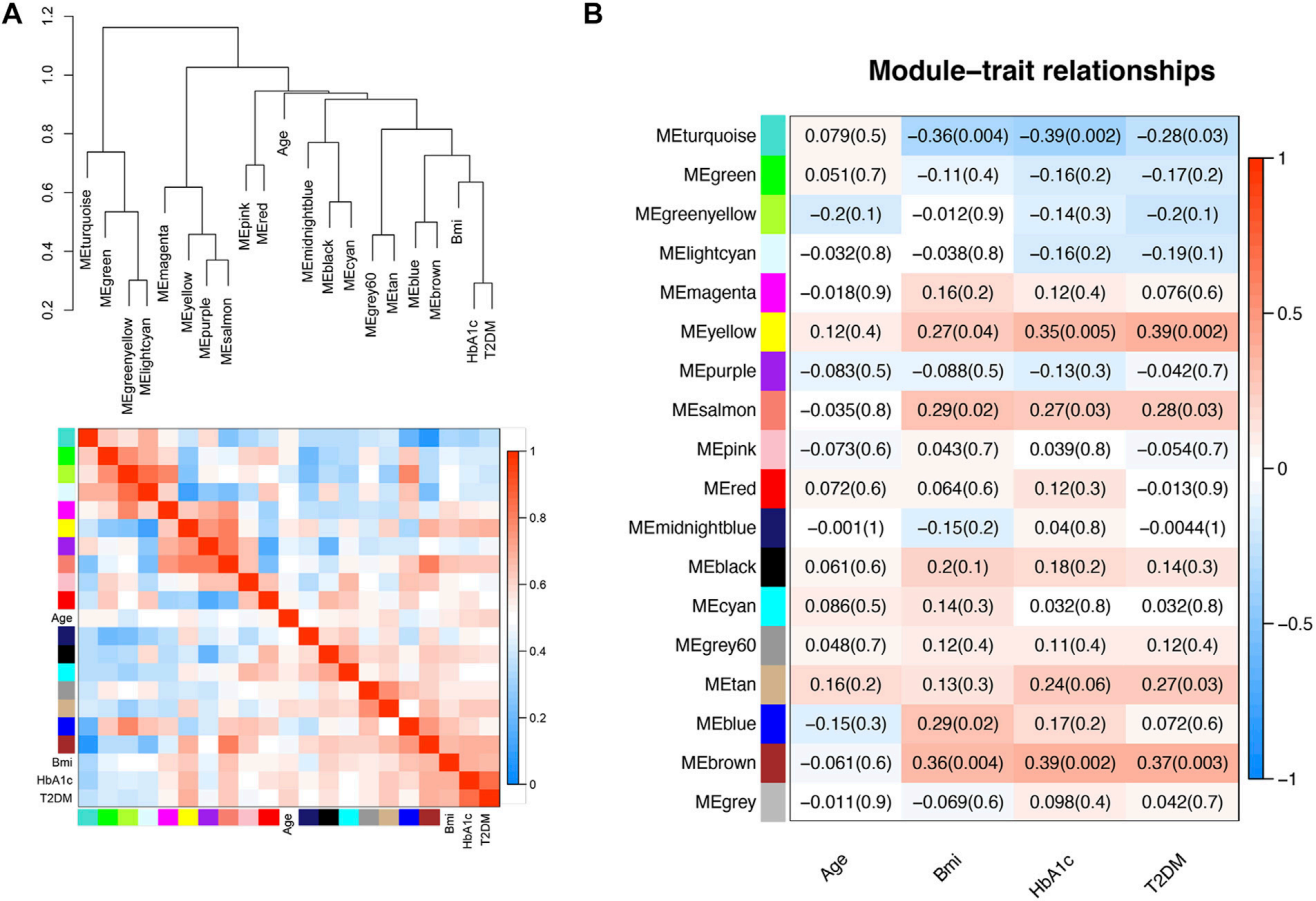
Relationships between the modules and clinical characteristics. **(A)** Hierarchical clustering dendrogram of MEs (marked with corresponding colors) and clinical characteristics. In the heatmap, red indicates high adjacency (positive correlation), and blue indicates low adjacency (negative correlation). **(B)** Module-trait relationships. Each column corresponds to a clinical trait, and each row corresponds to an ME. The numbers in the rectangles are the correlation coefficient and the *p*-value. Red indicates a positive correlation, and blue indicates a negative correlation.

The groups of correlated eigengenes were identified with a hierarchical clustering dendrogram and a heatmap (Figure 2A). In the present study, the clinical characteristics included age, BMI, HbA1c and disease state (T2DM). The main purpose of our study was to explore the potential pathogenesis of T2DM, so we selected the yellow module (R = 0.39, *p* = 0.002), which was most closely correlated with T2DM, for further analysis. The yellow module was also correlated with BMI (R = 0.27, *p* = 0.04) and HbA1c (R = 0.35, *p* = 0.005), but the correlation with HbA1c was more significant than that with BMI. There was no correlation with age (Figure 2B).

### Functional Enrichment Analysis

**FIGURE 3:**
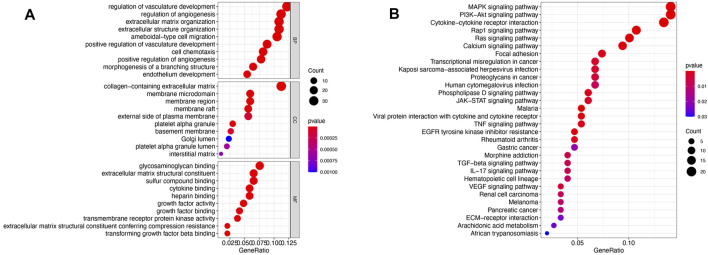
Functional enrichment of the yellow module. **(A)** Bubble plots of the results of GO enrichment analysis. The top 10 terms in the BP, CC, and MF categories with regard to the number of enriched genes in the yellow module as determined by GO analysis are shown. **(B)** Bubble plots of the KEGG pathways for the yellow module.

All genes in the yellow module were subjected to GO and KEGG analyses, and the top 10 terms based on the number of genes are shown in chord plots (Figure 3). The GO analysis results showed that the main enriched terms in the biological process (BP) category were the regulation of vasculature development, regulation of angiogenesis, extracellular structure organization, extracellular matrix organization, cell chemotaxis, and endothelium development terms, among others (Figure 3A). The enriched cellular component (CC) terms in the GO analysis were the collagen−containing extracellular matrix, membrane microdomain, and membrane region terms (Figure 3A). The main enriched molecular function (MF) terms in the GO analysis were the glycosaminoglycan binding, extracellular matrix structural constituent, cytokine binding, growth factor activity and transforming growth factor beta binding terms (Figure 3A). In KEGG pathway analysis, the genes in the yellow module were enriched mostly in the MAPK signaling pathway, PI3K−Akt signaling pathway, cytokine−cytokine receptor interaction pathway, Rap1 signaling pathway and Ras signaling pathway (Figure 3B).

### Identification of Hub Genes in the Yellow Module

**FIGURE 4:**
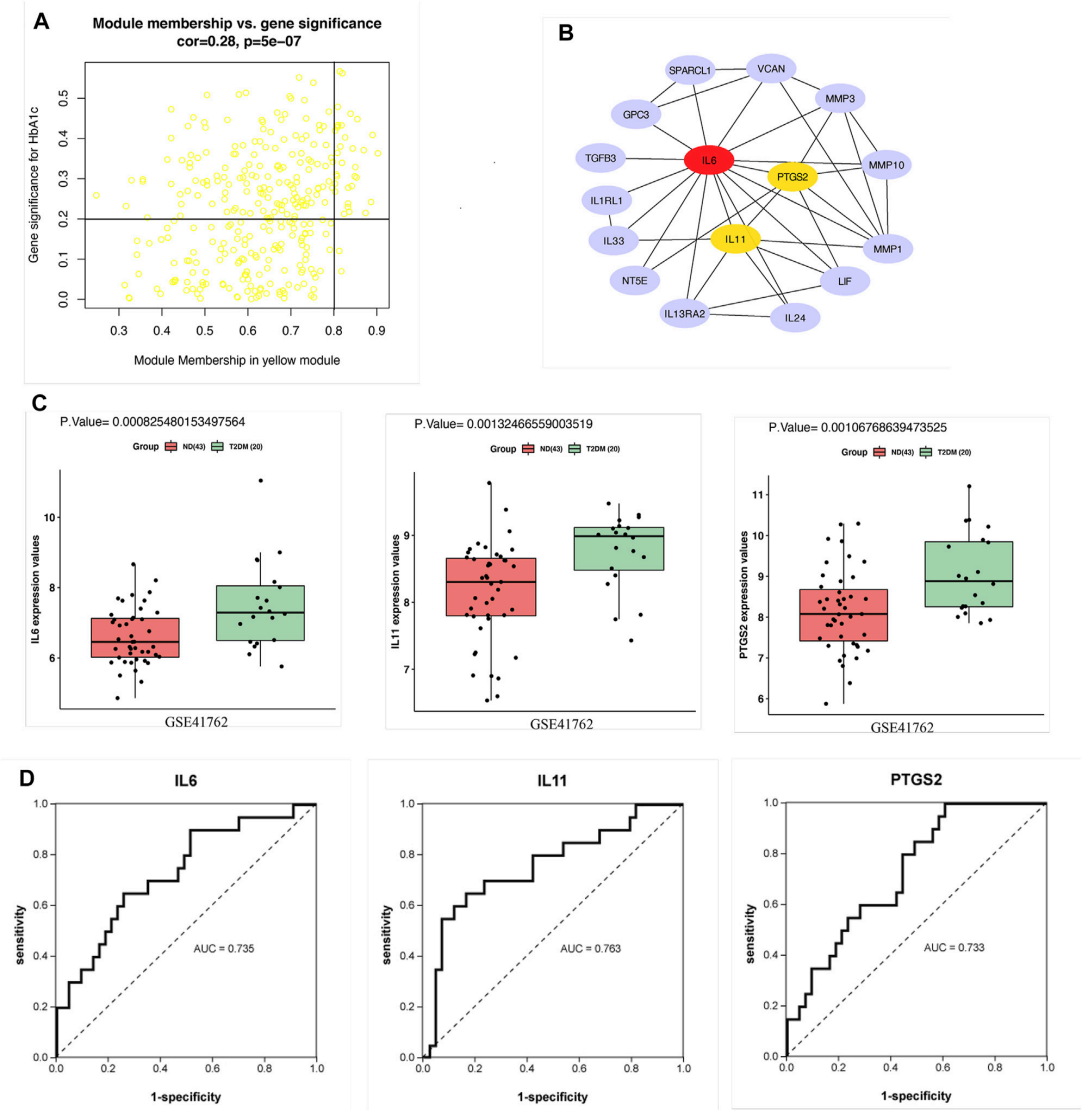
Hub gene screening. **(A)** Scatter plot of module eigengenes in the yellow module. **(B)** PPI network of 30 candidate hub genes in the yellow module. The hub genes were screened according to the degree of connectivity. A higher degree is represented by a darker color, which indicates that the genes are more central. The top genes were IL6, IL11 and PTGS2. **(C)** Expression of hub genes in GSE41762. **(D)** ROC curve analysis of the ability of IL6, IL11 and PTGS2 to distinguish between T2DM and ND samples. ROC curves were used to illustrate the diagnostic ability, sensitivity and specificity of the genes. Genes with AUC values >0.7 have certain diagnostic value. T-testing was performed to compare the means of the two groups.

Since the correlation between the yellow module and HbA1c was more significant than the correlation between the yellow module and BMI. Therefore, screening was conducted according to the thresholds of an IMMI≥0.8 and an IGSI≥0.2 for HbA1c, and a total of 30 genes that were closely connected to the yellow module were identified as candidate hub genes (Figure 4A, Supplementary Table S2). A PPI network for the candidate hub genes was constructed with the STRING database. There were 30 nodes and 45 edges in the PPI network, which represented the proteins and interactions, respectively (Figure 4B). A confidence score >0.4 was considered to indicate significance, and the top three genes with the highest degrees of connectivity [interleukin 6 (IL6), interleukin 11 (IL11) and prostaglandin-endoperoxide synthase 2 (PTGS2)] were selected as the hub genes (Supplementary Table S3). The gene expression levels of IL6 (*p* = 0.0008), IL11 (*p* = 0.001) and PTGS2 (*p* = 0.001) in the T2DM group were significantly higher than those in the ND group (Figure 4C). In addition, the AUCs of IL6, IL11 and PTGS2 were all greater than 0.7 in the ROC analysis, which was sufficient for discrimination of T2DM samples from ND samples (Figure 4D).

### Validation and Effectiveness Evaluation of the Hub Genes

**FIGURE 5:**
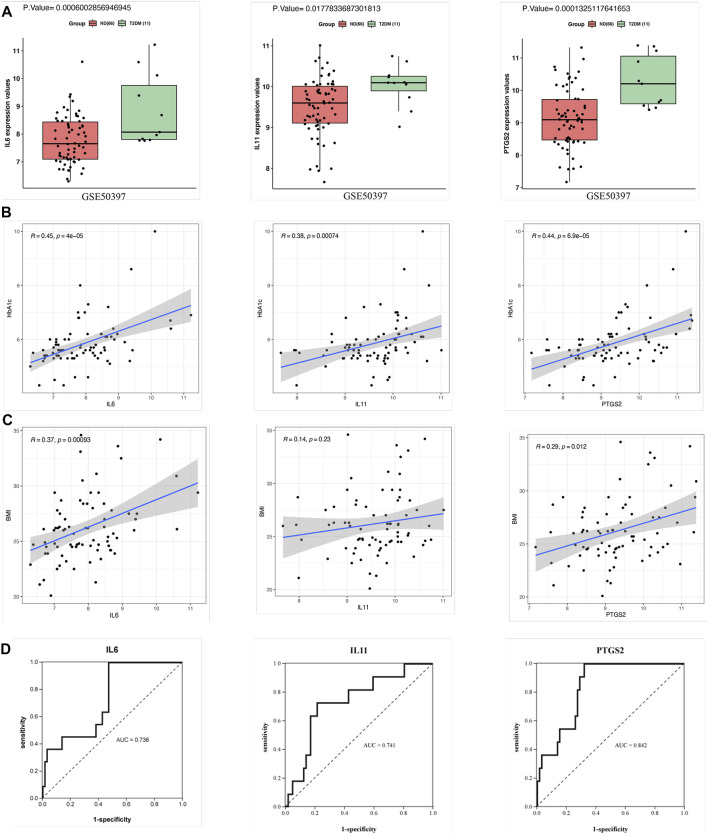
Validation of the hub genes in GSE50397. **(A)** Expression of the hub genes in human islets. The gene expression of IL6, IL11, and PTGS2 in the T2DM group was significantly greater than that in the ND group. **(B)** Correlation analysis between the hub genes and HbA1c. IL6, IL11, and PTGS2 all showed significant positive correlations with HbA1c. **(C)** Correlation analysis between the hub genes and BMI. The results showed that IL6 and PTGS2 were positively correlated with BMI but that IL11 was not significantly correlated with BMI. **(D)** ROC curve analysis of the hub genes. T-testing was performed to compare the means of the two groups.

The expression patterns of the hub genes were verified using the GSE50397 dataset. According to the recommendation of the International Expert Committee of the American Diabetes Association for the diagnosis of diabetes, an HbA1c level ≥6.5% was used as the threshold for T2DM (American Diabetes Association., 2019). Therefore, GSE50397 contained 11 T2DM samples and 66 ND samples (Supplementary Table S4). IL6 (*p* = 0.0006), IL11 (*p* = 0.01) and PTGS2 (*p* = 0.0001) were significantly upregulated in the T2DM samples (Figure 5A). Furthermore, IL6 (R = 0.45, *p* = 0.00004), IL11 (R = 0.38, *p* = 0.0007) and PTGS2 (R = 0.44, *p* = 0.00006) were all significantly positively correlated with HbA1c (Figure 5B), and IL6 (R = 0.37, *p* = 0.0009) and PTGS2 (R = 0.29, *p* = 0.01) were also positively correlated with BMI, but there was no correlation between IL11 and BMI (Figure 5C). ROC analysis showed that the AUCs of the above hub genes were all greater than 0.7, which showed that the three have certain value for distinguishing T2DM samples from ND samples (Figure 5D).

### Rat Experiments

#### HFD Rat Model

**FIGURE 6:**
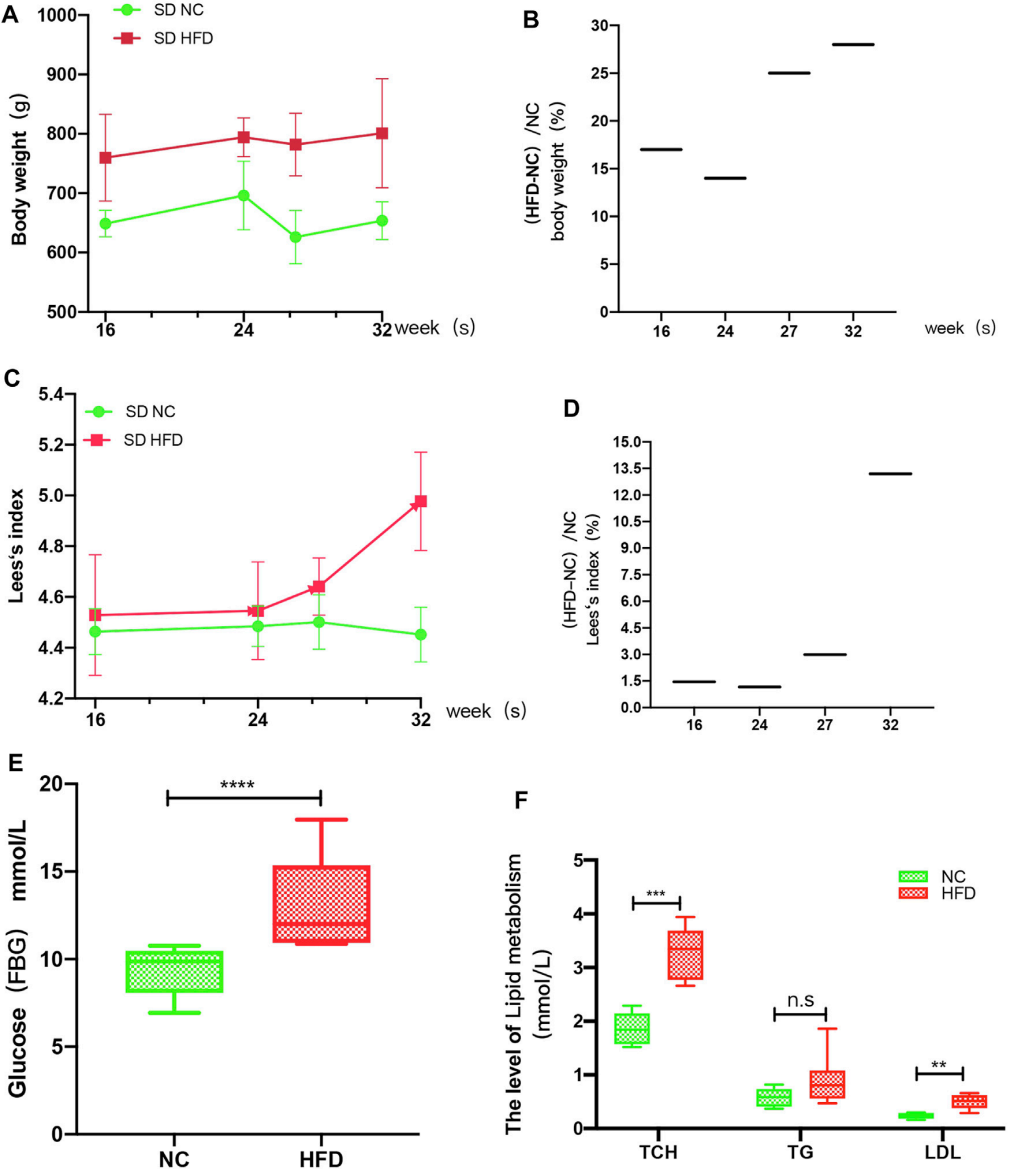
HFD rat model. The rats’ weights, body lengths, FBG levels and blood lipid (TG, TCH, and LDL) levels were measured at 16, 24, 26, and 32 weeks. **(A)** The weight gain of the HFD rats at 16, 24, 27, and 32 weeks was significantly greater than that of the NC rats. **(B)** The percent weight gain of the HFD rats at 16, 24, 27, and 32 weeks was 17, 14, 25 and 28%, respectively. **(C)** Lee’s index of the rats. **(D)** The Lee’s index values were increased by 1.457, 1.16, 2.99, and 13.2% at 16, 24, 27, and 32 weeks, respectively. **(E)** At 27 weeks, the FBG levels were significantly higher in the HFD rats than in the NC rats. **(F)** At 27 weeks, the TCH and LDL levels of the HFD group were significantly greater than those of the NC group, while there was no significant difference in TG level between the groups. The results are presented as the mean ± standard deviation (*t*-test; *n* = 6 in each group). *p < 0.05,**p < 0.01,***p < 0.001,****p < 0.0001, n. s., not significant.

The FBG levels, lipid (TG, TCH, LDL) levels, body lengths and body weights of the rats were measured at 16, 24, 27, and 32 weeks. The results showed that the body weights of HFD rats were more than 15% higher than those of the NC rats at weeks 16, 27, and 32 (Figures 6A,B), while smaller differences were observed for the Lee’s index values: the values for the HFD rats were more than 1.5% higher than those for the NC rats at weeks 27 and 32 (Figures 6C,D). Therefore, the HFD models were successfully constructed at 27 and 32 weeks. Analysis of the biochemical parameters of the 27-week-old SD rats showed that the FBG (*p <* 0.0001), TCH (*p* = 0.0002) and LDL (*p* = 0.001) were all significantly higher in the HFD rats than in the NC rats, while TG levels did not differ significantly between the groups (Figures 6E,F).

#### Expression Patterns of the Hub Genes in the Islets of HFD-Fed Rats

**FIGURE 7:**
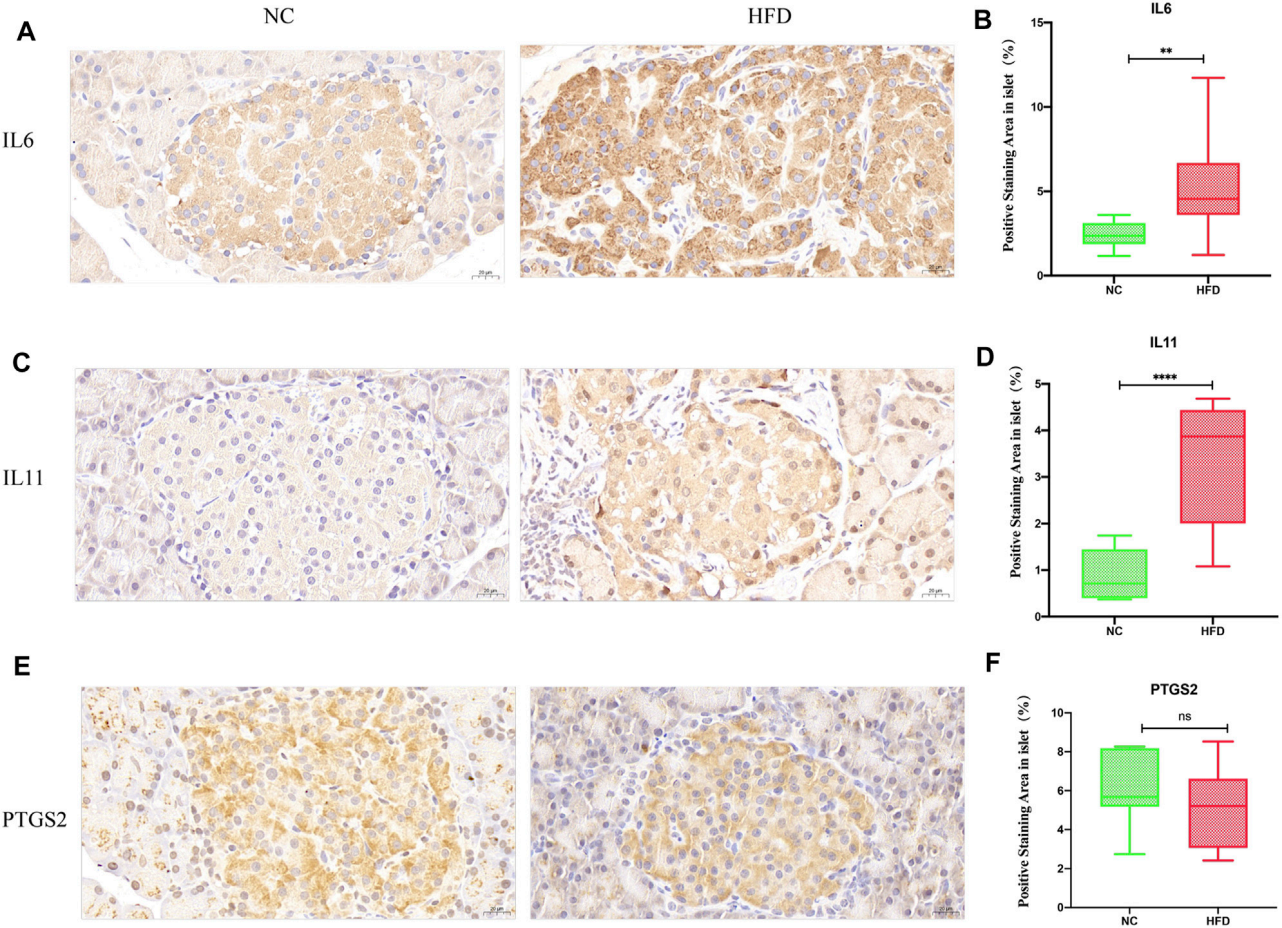
Expression of the hub genes in islets. IL6, IL11 and PTGS2 were all cytoplasmic. No staining or weakly positive staining was defined as low expression, and strong positive staining was defined as high expression. The density of positive staining was calculated by dividing the area of brown-stained structures within the islet parenchyma by the islet area. Quantifications were performed in a blind fashion. **(A,B)** Immunohistochemical staining showed that IL6 positivity was greater in the HFD group than in the NC group (40× magnification; scale bar: 20 µm). **(C,D)** Immunohistochemical staining showed that IL11 positive staining was greater in the HFD group than in the NC group (40× magnification; scale bar: 20 µm). **(E,F)** There was no significant difference between the HFD group and the NC group with regard to PTGS2 immunohistochemical staining (40× magnification; scale bar: 20 µm). The results are presented as the mean ± standard deviation. (*t*-test; n = 6 in each group). *p < 0.05,**p < 0.01,***p < 0.001,****p < 0.0001, n. s., not significant.

We examined whether HFD feeding affects the expression of IL6, IL11, and PTGS2 by immunohistochemistry. According to a visual grading system, weak positive and negative staining were defined as low expression, and strong positive staining was defined as high expression. We used ImageJ to performed the quantification for the area of positive staining. The results showed that the expression level of IL6 (*p* = 0.01) and IL11 (*p* < 0.0001) was higher in the HFD group than that in the NC group (Figures 7A,B,C,D), but PTGS2 (*p* = 0.5) expression level didn’t show significant change between the HFD group and the NC group (Figure 7E,F).

#### Fibrosis of Islets Under HFD Feeding

**FIGURE 8:**
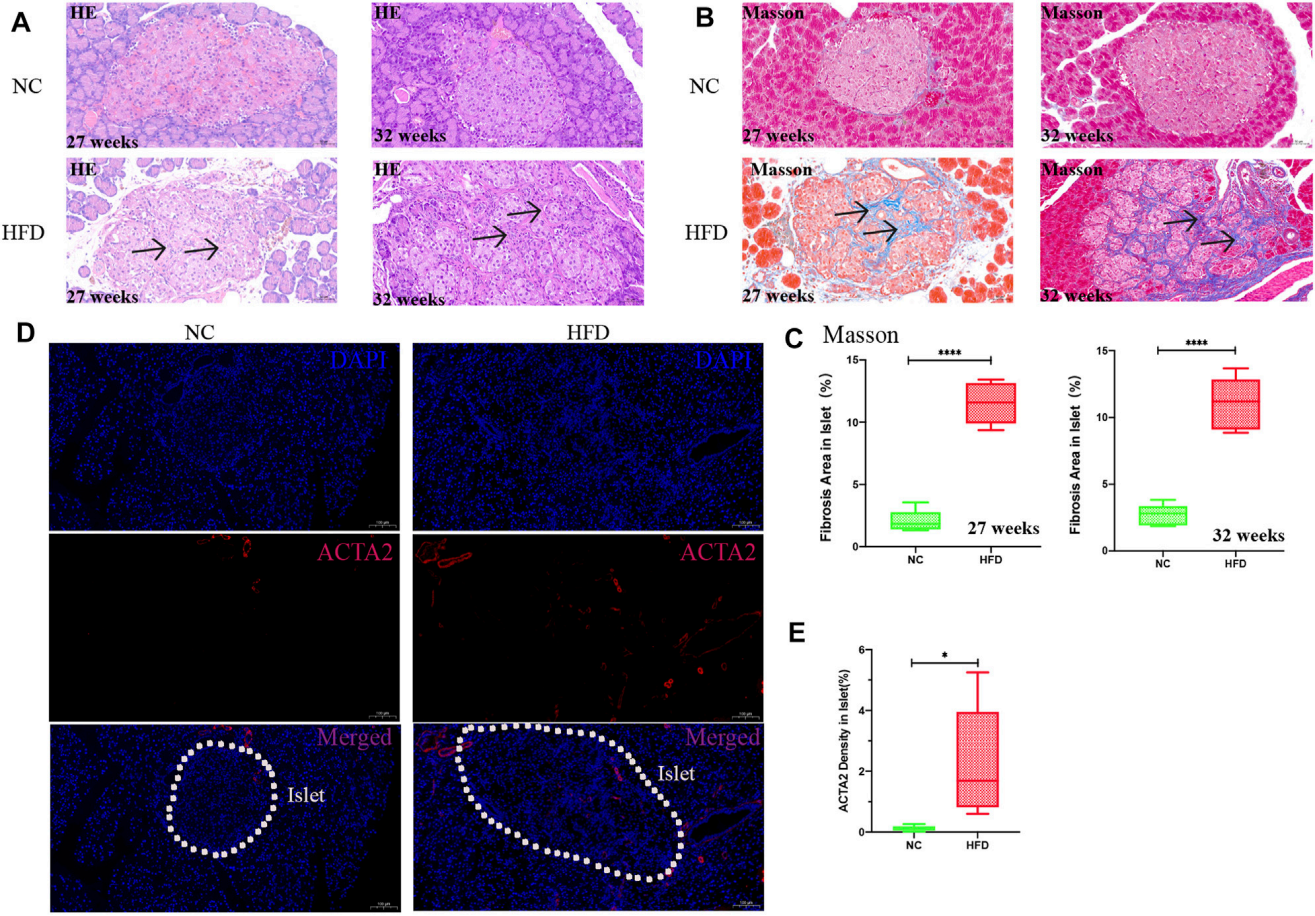
Pathology and fibrotic protein detection in islets. H&E staining and Masson staining were used to observe the islet tissue, and ACTA2 was detected by immunofluorescence. The percentage of fibrosis was calculated by dividing the area of blue fiber within the islet parenchyma by the islet area, and the density of the ACTA2 was calculated by dividing the area of red fluorescence within the islet by the islet area. Quantification was performed in a blind fashion.**(A)** H&E staining in islets at 27 and 32 weeks (20× magnification; scale bar: 50 µm). After HFD intervention, the area of islets varied and were mostly large or medium in size, with irregular edges and a large amount of fibrosis in the islet interstitium (*arrows*). The islets were divided into medium-sized clumps, and the cells in the islets were arranged in disorder. **(B,C)** Masson staining (20× magnification; scale bar: 50 µm). A large amount of collagen fibrin was visible in the islets after HFD intervention (*arrows*). **(D,E)** Immunofluorescence analysis after 27 weeks of HFD intervention. The expression of ACTA2 (red) in islets as determined by immunofluorescence staining was significantly higher in the HFD group than in the NC group (10× magnification; scale bar: 100 µm). The results are presented as the mean ± standard deviation (*t*-test; n = 6 in each group). *p < 0.05,**p < 0.01,***p < 0.001,****p < 0.0001, n. s., not significant.

The GO functional enrichment analysis of the yellow module genes revealed that they were closely related to the synthesis of extracellular matrix collagen, and the expression of fibrosis-related genes [ACTA2, decorin (DCN) and cartilage oligomeric matrix protein (COMP)] was significantly higher than that in the ND group (Supplementary Figure S1). Thus, H&E staining and Masson staining were performed on pancreatic tissues. The results showed that there was substantial fibrosis in the islets of HFD rats, in addition, the structure of the islets was destroyed, the islets were divided into multiple small clumps, and the cell arrangement in the islets was disordered (Figures 8A,B). Quantitative analysis of Masson staining at 27 weeks (*p* < 0.0001) and at 32 weeks (*p* < 0.0001) showed that the islets of HFD rats had more fibrosis (Figure 8C). Immunofluorescence showed that the level of the fibrotic protein ACTA2 (*p* = 0.03) was somewhat higher in the HFD group than in the NC group (Figure 8D,E).

## Discussion

Obesity (BMI ≥28 kg/m^2^) can cause unusually high levels of lipids (Cong et al., 2014; Zheng et al., 2018), and HbA1c is a good indicator of fluctuations in a patient’s blood glucose during the past 3 months (Monnier et al., 2003; Giugliano et al., 2008). Therefore, we sought to explore the potential pathogenesis of islet dysfunction induced by high glucose and lipids by using WGCNA to search for gene modules related to BMI and HbA1c. In this study, we found that the gene module most closely related to T2DM was also significantly positively related to HbA1c. In addition, this gene module was involved in the inflammatory response and in synthesis of extracellular matrix collagen, and the hub genes IL6 and IL11 play important roles in inducing inflammation and collagen synthesis. Therefore, we speculate that inflammation and fibrosis induced by glucotoxicity-activated IL6 and IL11 might be an important mechanism of islet dysfunction.

Glucotoxicity exists in T2DM and has a negative effect on islet function (Weir, 2020). In our study, the yellow module had the strongest correlation with T2DM and was significantly positively correlated with HbA1c, but it was only weakly correlated with BMI. Although glucotoxicity, lipotoxicity and glucolipotoxicity can all lead to islet dysfunction, the related mechanisms are not identical. Therefore, we speculated that changes in blood glucose might have a deeper impact on the genes than lipids in this module. The GO enrichment and KEGG pathway analyses showed that the yellow module genes were enriched mainly for the extracellular matrix collagen synthesis, collagen−containing extracellular matrix, cytokine binding and cytokine−cytokine receptor interaction terms, among others. These findings are similar to those of previous studies reporting that glucotoxicity can affect β-cell calcium ion exchange, endoplasmic reticulum stress, mitochondrial disorders, and oxidative stress through inflammation or direct stimulation (Chen, et al., 2008; Dingreville, et al., 2019; Ježek et al., 2019). However, the effect of glucotoxicity on extracellular matrix collagen has rarely been reported. Secretion of extracellular matrix proteins is a defining feature of fibrosis (Wynn, 2008). A large amount of extracellular matrix collagen in tissues and organs is produced by mesenchymal cells (such as pericytes and myofibroblasts), and endothelial/epithelial cells transform into myofibroblasts when stimulated by active soluble mediators (cytokines, chemokines, and growth factors), which eventually leads to the destruction of parenchymal cells (Weiskirchen, et al., 2019). In addition to finding enriched GO terms related to fibrosis, we found that the fibrotic proteins ACTA2, DCN, and COMP were all significantly upregulated in GSE41762 (Supplementary Figure S1). Furthermore, we observed significant extracellular matrix collagen deposition and islet structure destruction in our HFD-induced T2DM rat model. These changes were accompanied by disordered arrangement and decreased numbers of β cells and by increased fibrin ACTA2 levels in islets. These data confirmed that islets underwent fibrotic changes in the HFD-induced T2DM rat model. Proper arrangement of β cells within islets is also critical for insulin release through generation of rhythmic activity (Johnston, et al., 2016). Thus, the effect of islet fibrosis on β cell function and activity cannot be ignored.

Inflammation is an important trigger for fibrosis (Mack, 2018). In the PPI network in this study, the cytokines IL6, interleukin 33 (IL33), transforming growth factor-β (TGF-β), and IL11, all of which are involved in fibrosis (Mahler, et al., 2013; Pérez, et al., 2017; Widjaja, et al., 2019), were significantly upregulated in T2DM (Supplementary Figure S2). Among them, IL6 and IL11 were the hub genes of this module. Both IL6 and IL11 belong to the IL6 family of cytokines (Lokau, et al., 2017), which plays an important role in inducing fibrosis (Mahler, et al., 2013; Widjaja, et al., 2019). Some reports have shown that IL6 can increase fibrosis by upregulating TGF-β to promote endothelial-to-mesenchymal transformation (EndMT) in endothelial cells (Mahler, et al., 2013) and can also maintain Th1 cell proliferation and interferon-γ (IFN-γ) secretion, thus reducing the degradation of matrix proteins (Fielding, et al., 2014). Additionally, IL11 has emerged as a master regulator of fibrosis and stromal inflammation (Cook and Schafer, 2020). IL11 is a less frequently studied cytokine that has only recently been shown to be an important downstream regulator of the responses to multiple diverse stimuli [such as TGF-β, basic fibroblast growth factor (bFGF), platelet-derived growth factor (PDGF), and angiotensin II (ANGII)] and is the nexus between profibrotic initiating factors and organ fibrosis (Widjaja, et al., 2019; Corden et al., 2020). IL6 and IL11 can activate the classic JAK/STAT, PI3K and MAPK pathways through gp130, and IL11 can also regulate ERK to initiate downstream inflammation and fibrosis (Widjaja, et al., 2019; Lokau, et al., 2017; Schafer, et al., 2017). Thus, IL6 and IL11 are key targets for inhibition of inflammation and fibrosis. In our study, IL6 and IL11 were not only significantly upregulated in T2DM samples but also positively correlated with HbA1c. In the rat experiment, the positive staining for IL6 and IL11 in islets was much stronger in rats with HFD-induced T2DM than in normal rats.

Based on the above findings, we speculate that high glucose-mediated increases in IL6 and IL11 with subsequent induction of inflammation and fibrosis in islets might be involved in an important pathogenic mechanism of islet dysfunction.

## Conclusion

This study had certain limitations. For example, islets are composed of β cells, α cells, PP cells, δ cells, endothelial cells, and other cell types (Chiou, et al., 2021); therefore, it is necessary to clarify which cells express IL6 and IL11. In addition, the mechanisms of IL6 and IL11 should be further clarified with *in vivo* and *in vitro* experiments. Despite the limitations, we drew some meaningful conclusions. Through bioinformatics analysis and animal experiments, we found that glucotoxicity may be an important factor inducing IL6 and IL11 upregulation, and we found that the fibrosis induced by IL6 and IL11 may be involved in the pathogenesis of islet dysfunction. Our findings contribute to the research foundation for targeted therapy of T2DM.

## Data Availability

Publicly available datasets were analyzed in this study. This data can be found here: https://www.ncbi.nlm.nih.gov/geo/query/acc.cgi?acc=GSE41762; https://www.ncbi.nlm.nih.gov/geo/query/acc.cgi?acc=GSE50397.
