# Supplementary figures and images for: Glucotoxicity Activation of IL6 and IL11 and Subsequent Induction of Fibrosis May Be Involved in the Pathogenesis of Islet Dysfunction
Source: Front Mol Biosci. 2021 Aug 23;8:708127. doi: 10.3389/fmolb.2021.708127 (PMC8419433; doi:10.3389/fmolb.2021.708127)

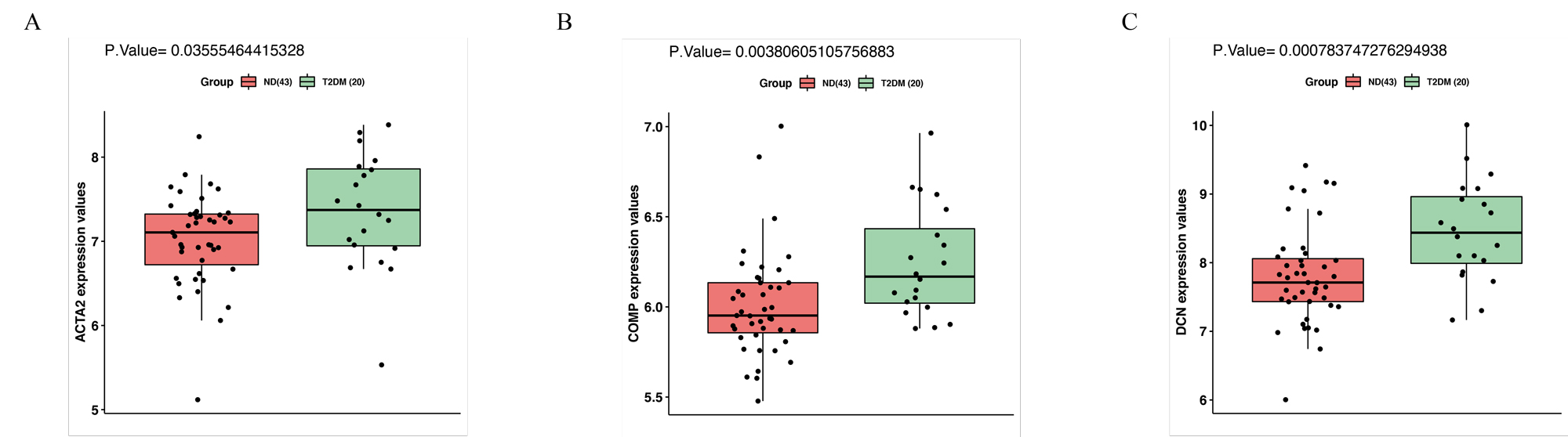

Supplement: Supplementary file 3 [file Image1.JPEG]

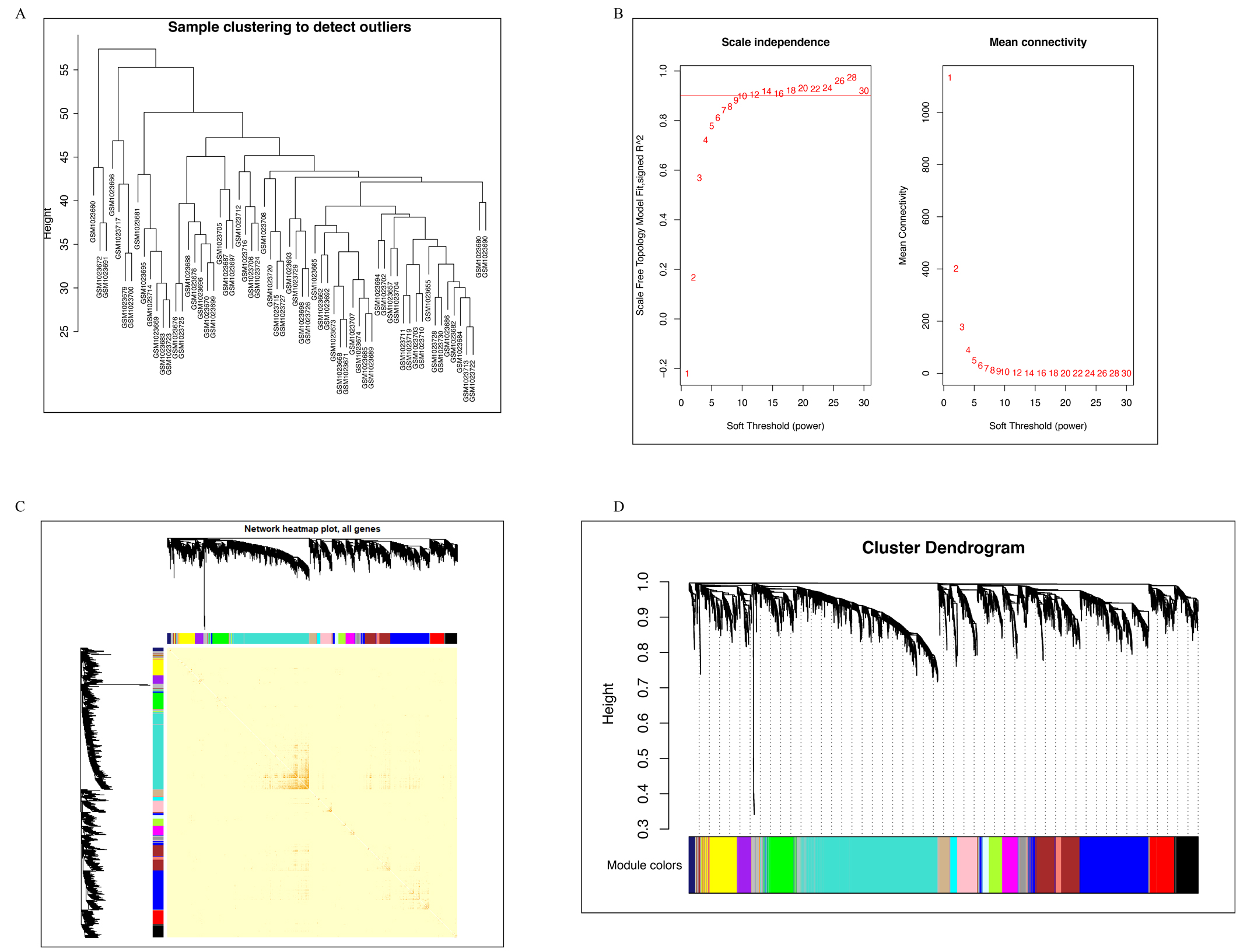

Supplement: Supplementary file 4 [file DataSheet1.ZIP › Supplementary Material Presentation/Figure 1.jpg]

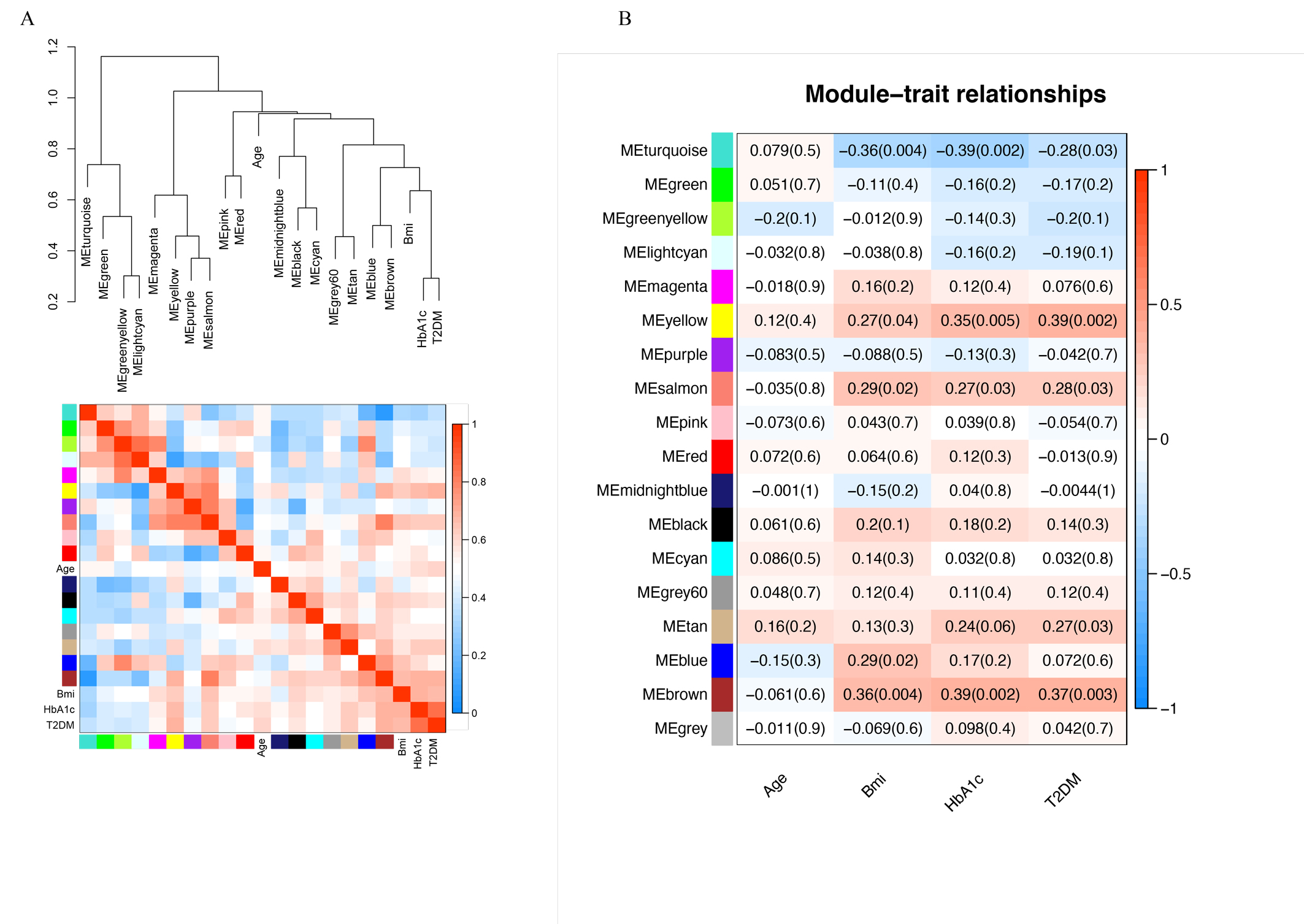

Supplement: Supplementary file 4 [file DataSheet1.ZIP › Supplementary Material Presentation/Figure 2.jpg]

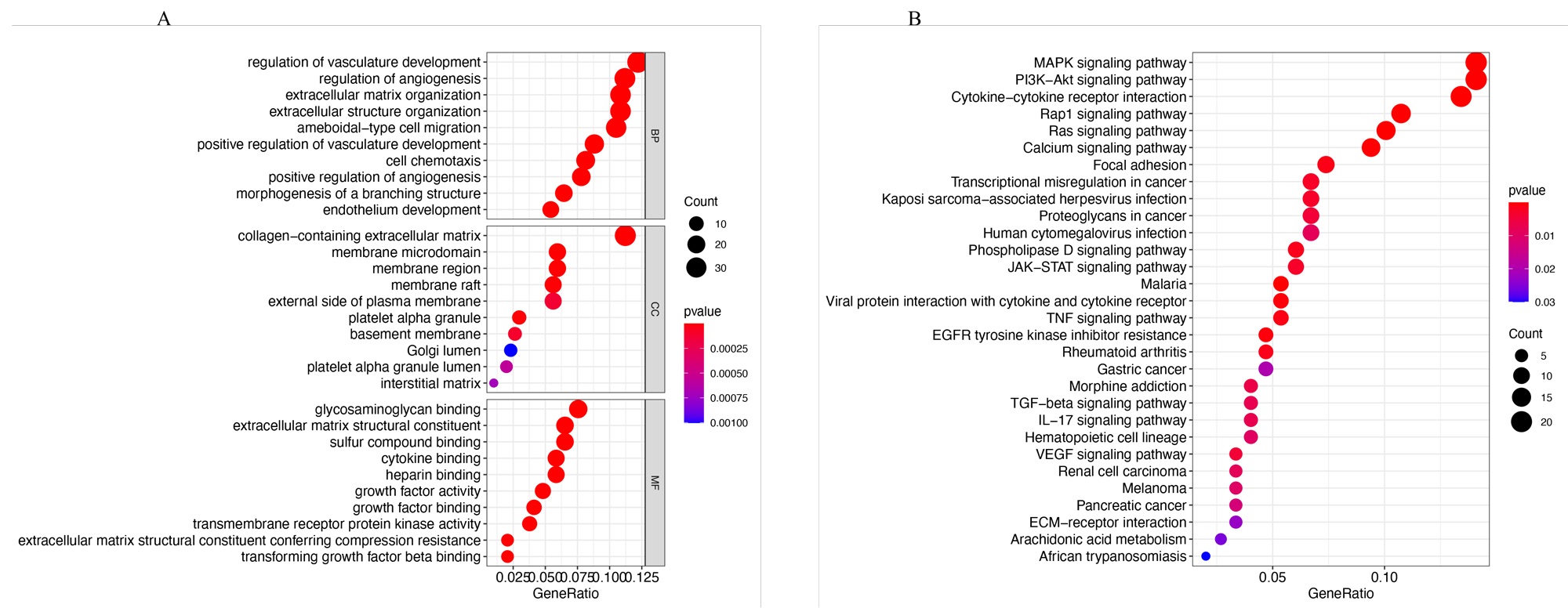

Supplement: Supplementary file 4 [file DataSheet1.ZIP › Supplementary Material Presentation/Figure 3.jpg]

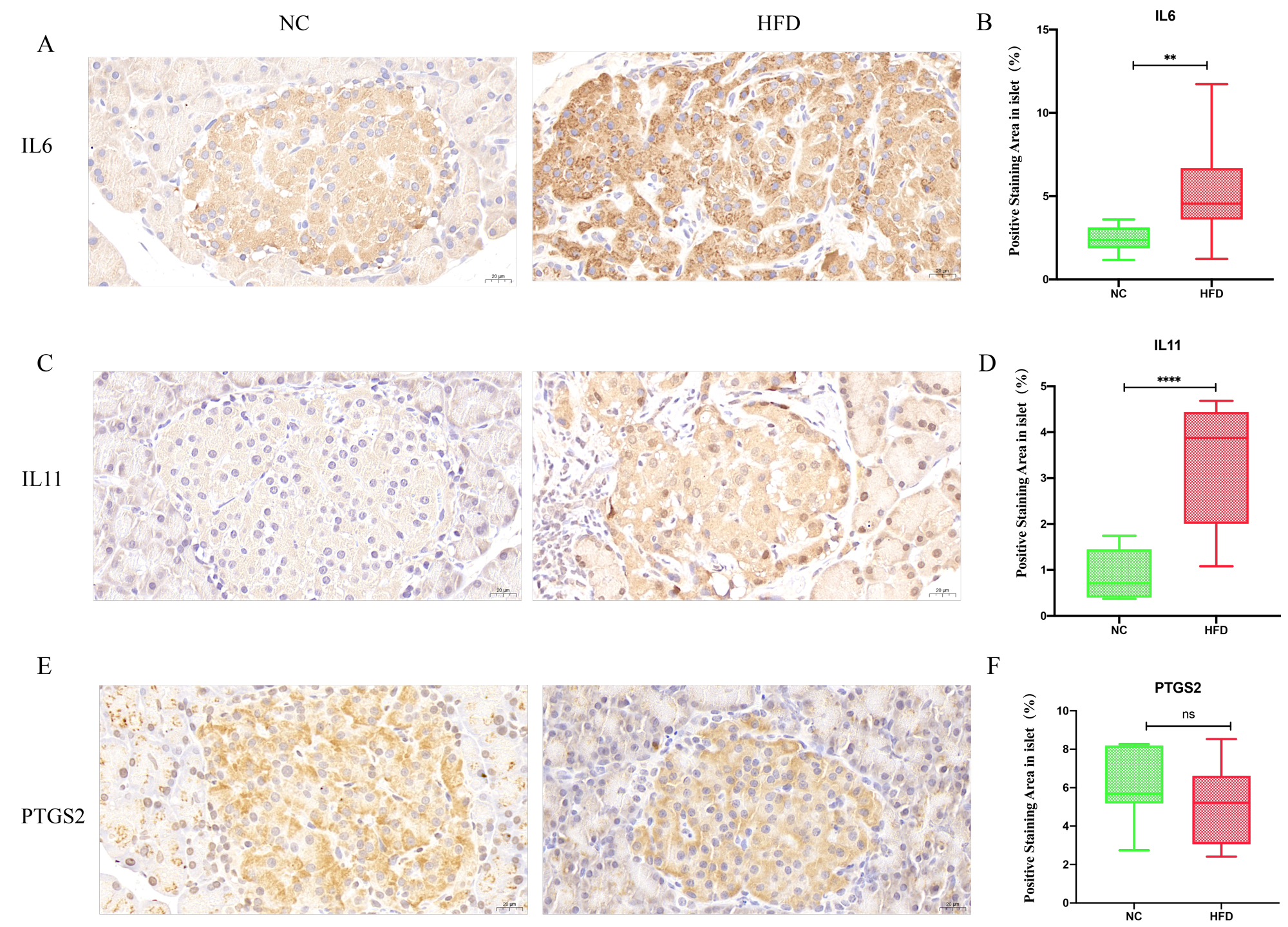

Supplement: Supplementary file 4 [file DataSheet1.ZIP › Supplementary Material Presentation/Figure 7.jpg]

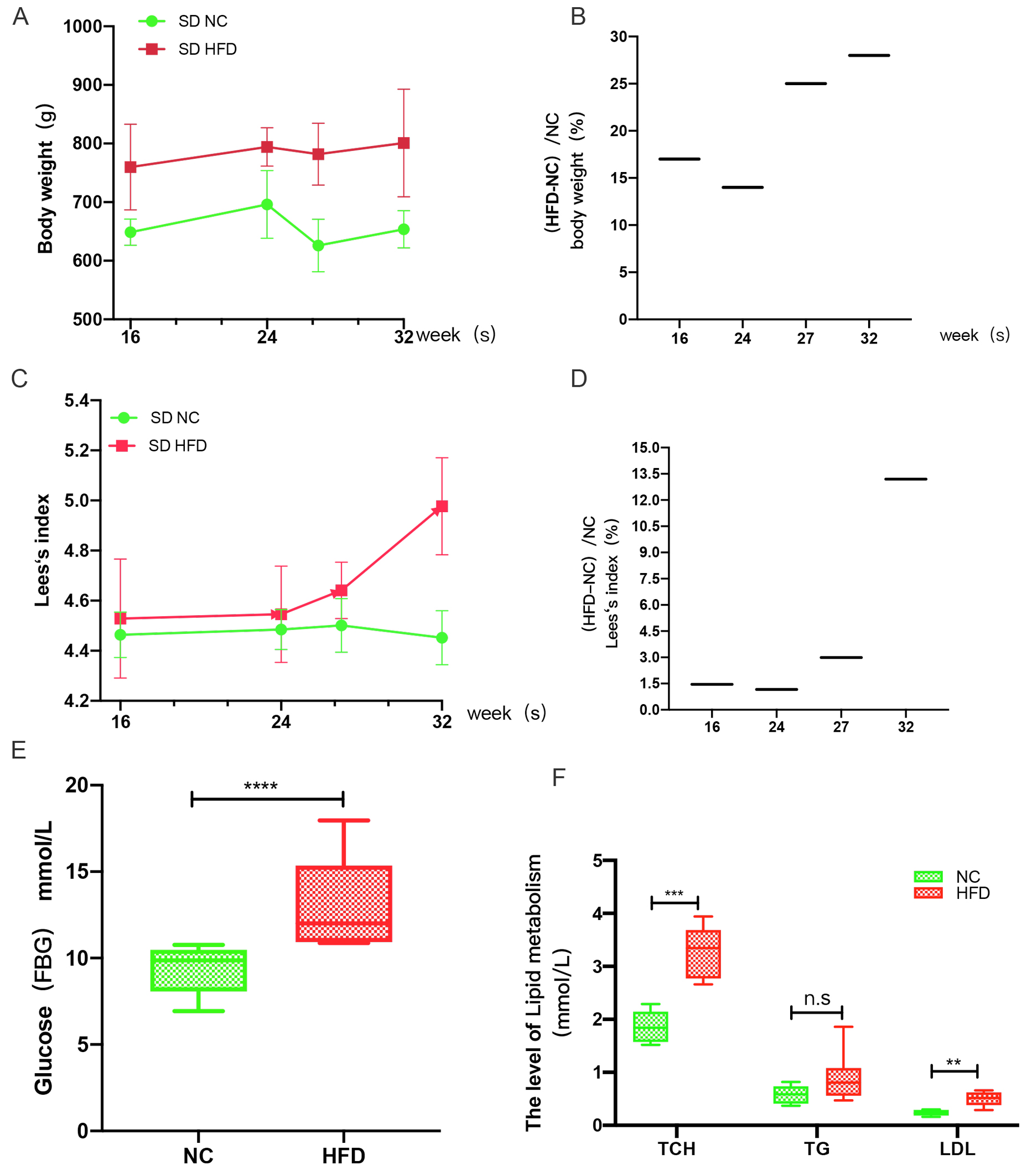

Supplement: Supplementary file 4 [file DataSheet1.ZIP › Supplementary Material Presentation/Figure 6.jpg]

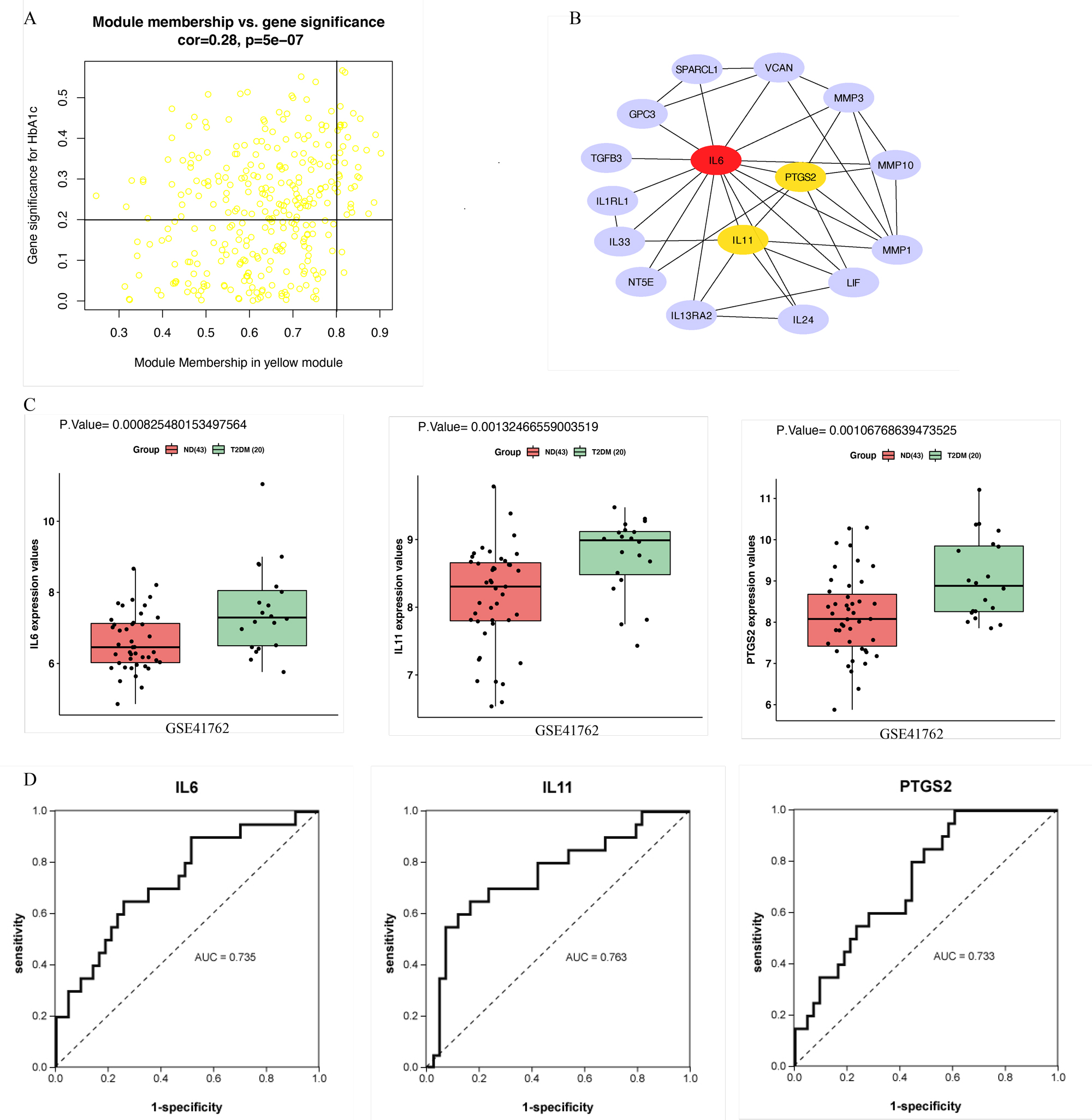

Supplement: Supplementary file 4 [file DataSheet1.ZIP › Supplementary Material Presentation/Figure 4.jpg]

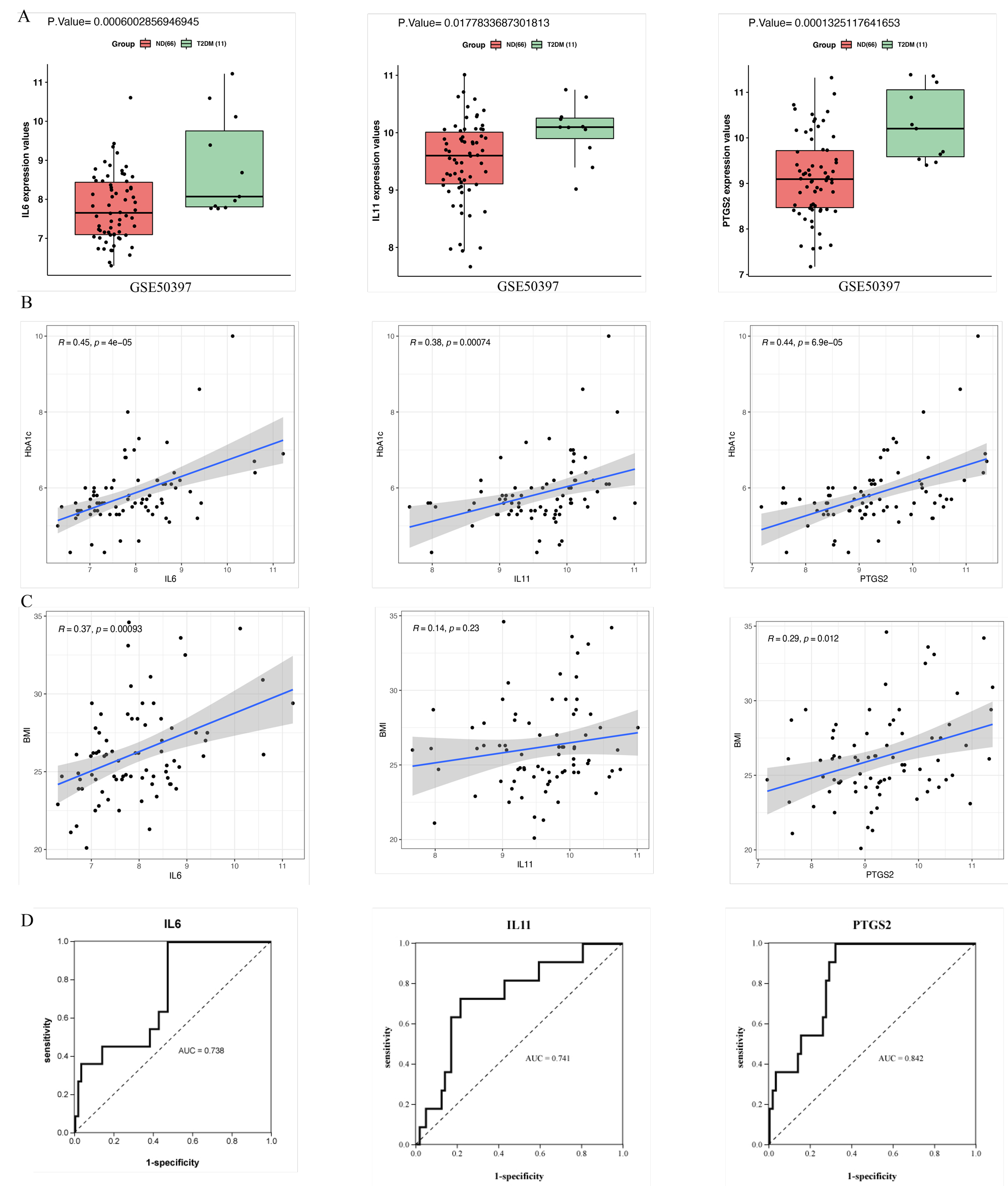

Supplement: Supplementary file 4 [file DataSheet1.ZIP › Supplementary Material Presentation/Figure 5.jpg]

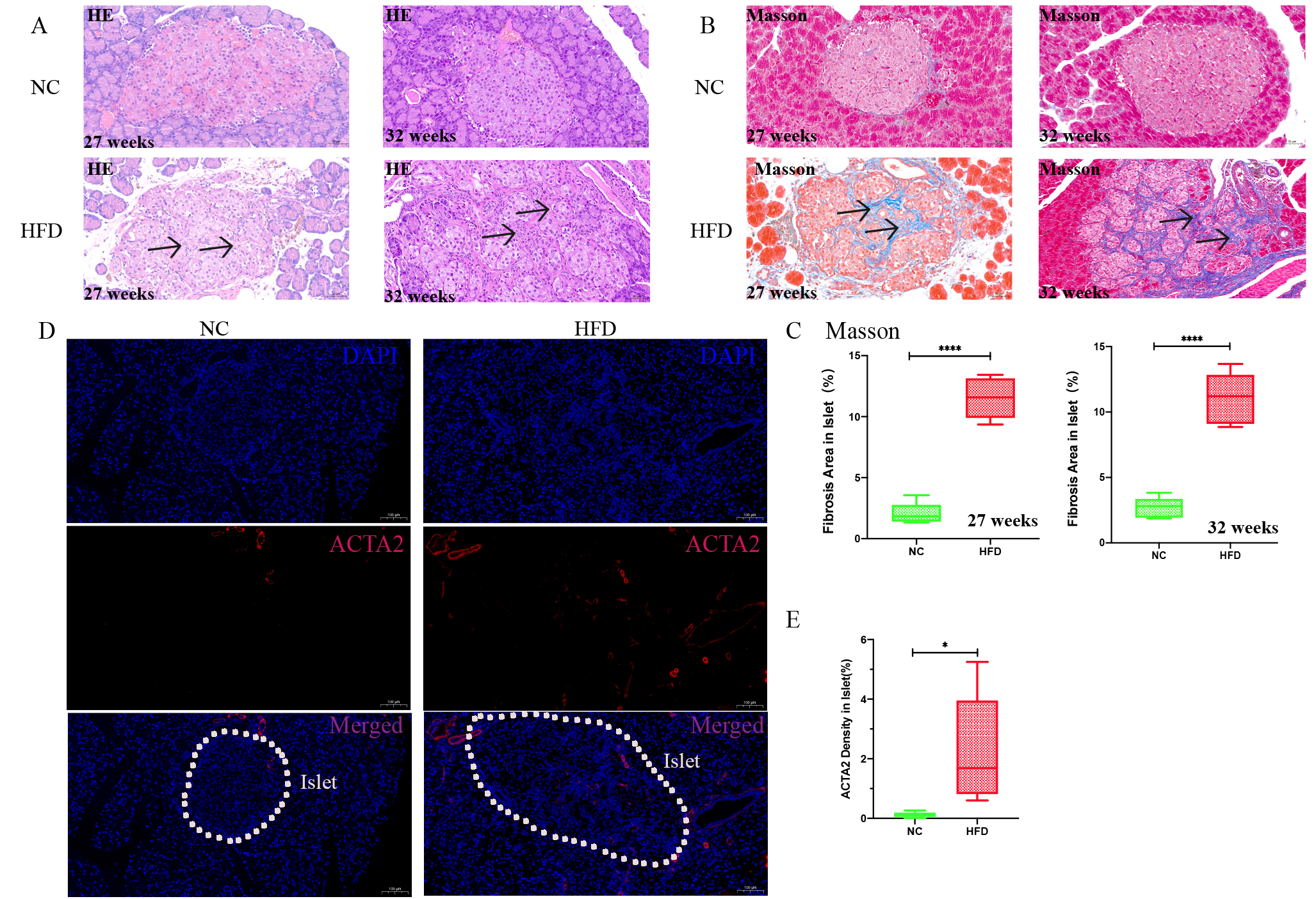

Supplement: Supplementary file 4 [file DataSheet1.ZIP › Supplementary Material Presentation/Figure 8.jpg]

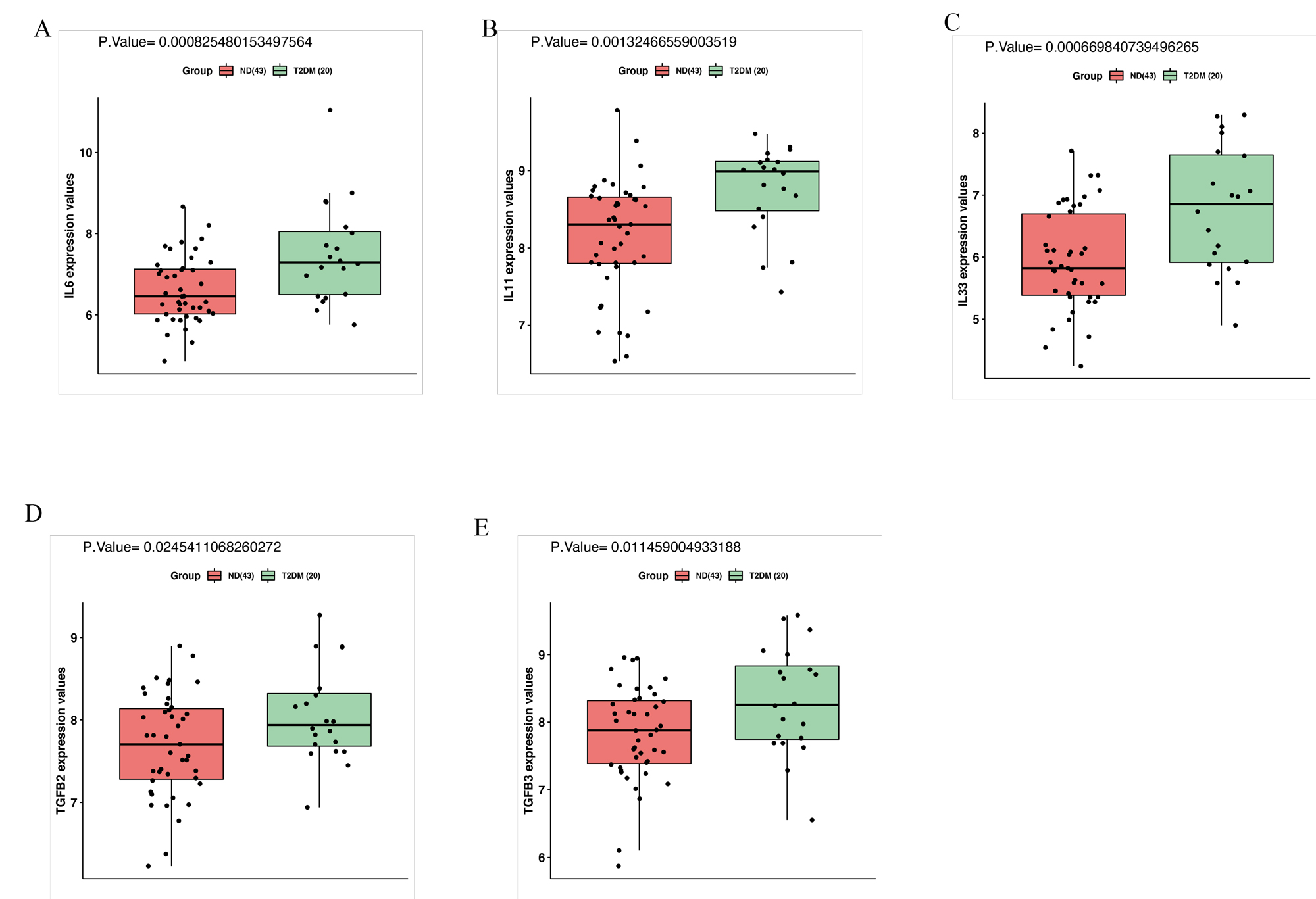

Supplement: Supplementary file 5 [file Image2.JPEG]
