# Supplementary material for: Glucotoxicity Activation of IL6 and IL11 and Subsequent Induction of Fibrosis May Be Involved in the Pathogenesis of Islet Dysfunction
Source: Front Mol Biosci. 2021 Aug 23;8:708127. doi: 10.3389/fmolb.2021.708127 (PMC8419433; doi:10.3389/fmolb.2021.708127)
Supplement: Supplementary file 6 [file Table5.DOCX]

Supplementary Material

# Supplementary Figures


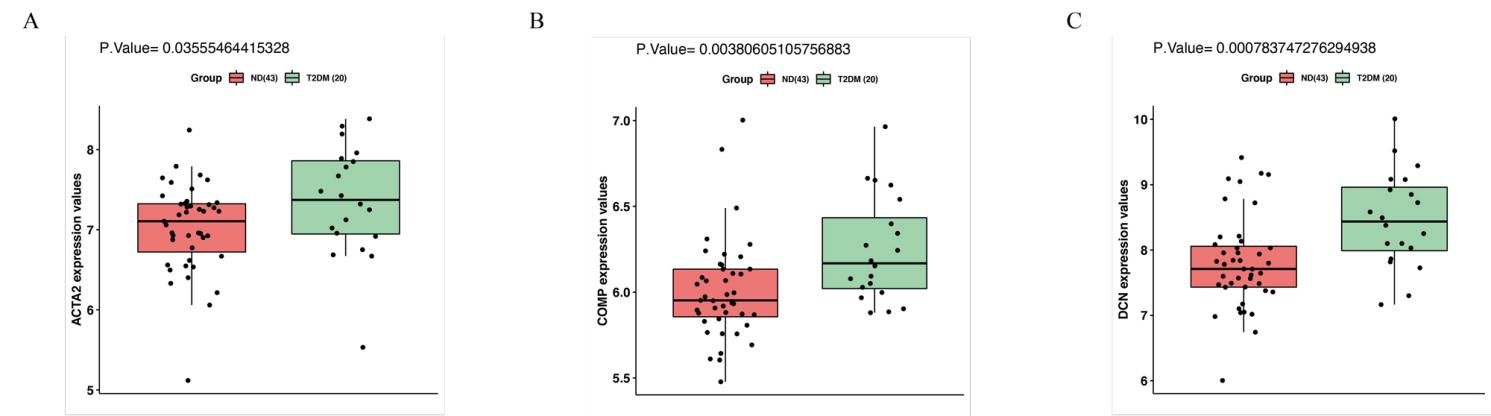


**Supplementary Figure 1** Expression of fibrosis-related genes in GSE41762. The fibrosis-related genes ACTA2 (A), COMP (B) and DCN (C) were significantly upregulated in the T2DM group. T-testing was performed to compare the means of the two groups.


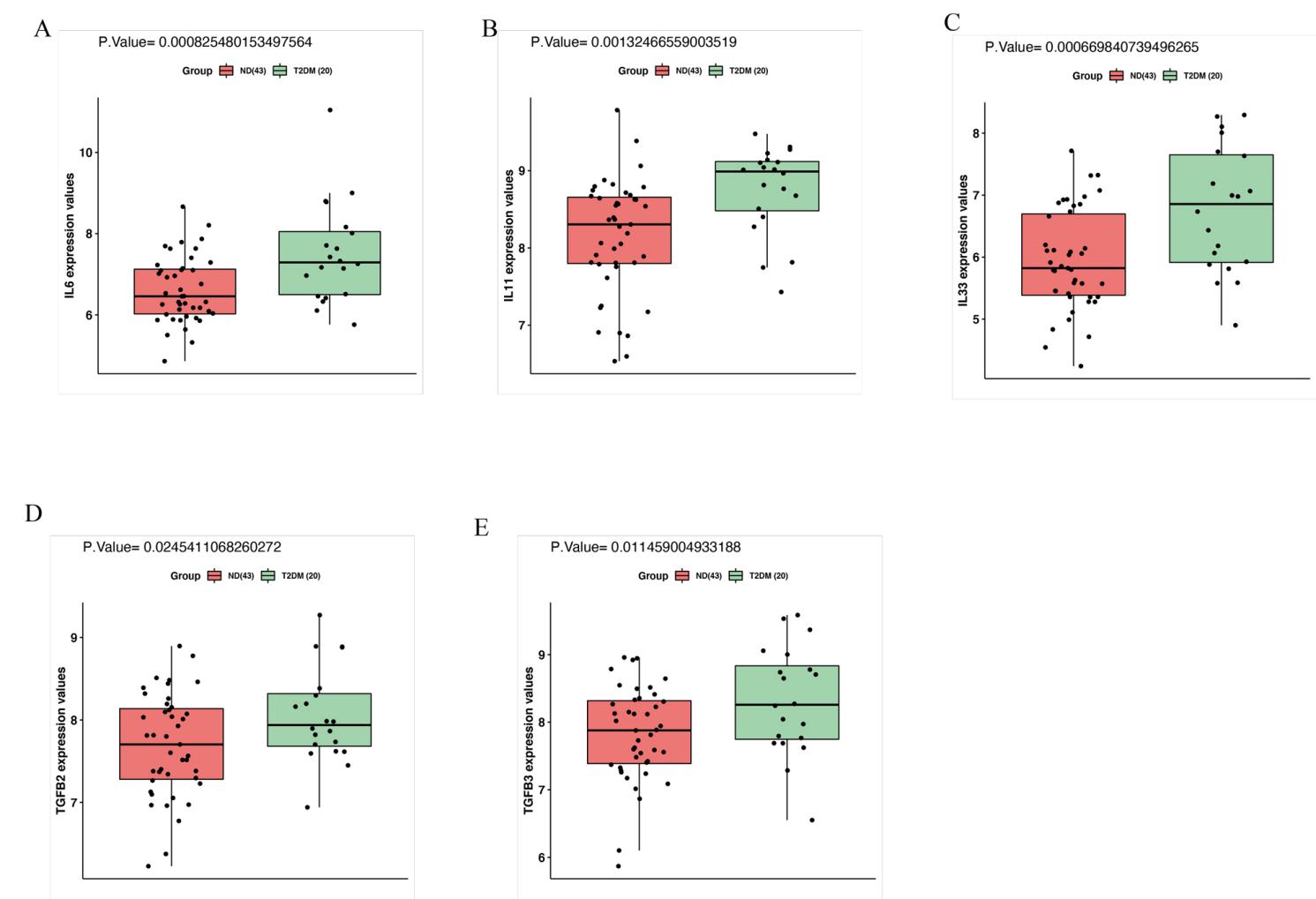


**Supplementary Figure 2** Expression of inflammation genes related to promotion of fibrosis in GSE41762. The results showed that the profibrotic factors IL6 (A), IL11 (B), IL33 (C), TGFβ2 (D) and TGFβ3 (E) were all significantly upregulated in the T2DM group. T-testing was performed to compare the means of the two groups.
